# Bidirectional convolutional LSTM for the prediction of nitrogen dioxide in the city of Madrid

**DOI:** 10.1371/journal.pone.0269295

**Published:** 2022-06-01

**Authors:** Ditsuhi Iskandaryan, Francisco Ramos, Sergio Trilles

**Affiliations:** Institute of New Imaging Technologies (INIT), Universitat Jaume I, Castelló de la Plana, Castellón, Spain; Hanyang University, KOREA, REPUBLIC OF

## Abstract

Nitrogen dioxide is one of the pollutants with the most significant health effects. Advanced information on its concentration in the air can help to monitor and control further consequences more effectively, while also making it easier to apply preventive and mitigating measures. Machine learning technologies with available methods and capabilities, combined with the geospatial dimension, can perform predictive analyses with higher accuracy and, as a result, can serve as a supportive tool for productive management. One of the most advanced machine learning algorithms, Bidirectional convolutional LSTM, is being used in ongoing work to predict the concentration of nitrogen dioxide. The model has been validated to perform more accurate spatiotemporal analysis based on the integration of temporal and geospatial factors. The analysis was carried out according to two scenarios developed on the basis of selected features using data from the city of Madrid for the periods January-June 2019 and January-June 2020. Evaluation of the model’s performance was conducted using the Root Mean Square Error and the Mean Absolute Error which emphasises the superiority of the proposed model over the reference models. In addition, the significance of a feature selection technique providing improved accuracy was underlined. In terms of execution time, due to the complexity of the Bidirectional convolutional LSTM architecture, convergence and generalisation of the data took longer, resulting in the superiority of the reference models.

## 1 Introduction

The increase in the level of urbanisation, in addition to positive consequences, also causes some problems associated with environmental changes, one of which is the deterioration of air quality [1, 2]. According to the observations of the World Health Organisation (WHO), seven million deaths due to short-term and long-term exposure to air pollutants are recorded every year [3]. Regarding Spain, studies show that over 93,000 people have died in Spain due to air pollution in recent decades [4]. The WHO has identified the most dangerous pollutants and established guidelines with specific thresholds for each of them, including particulate matter (PM), ozone (O_3_), nitrogen dioxide (NO_2_) and sulphur dioxide (SO_2_) [5, 6]. The prediction of one of these pollutants, NO_2_, is the main focus of the current work. The main source of NO_2_ formation is the combustion of fossil fuels, especially that produced by traffic. There are many works devoted to the study of the effects of NO_2_, in particular, the increase in mortality from cardiovascular and respiratory diseases. For example, Faustini et al. found out that the effect of an increase in the annual concentration of NO_2_ by 10 *μ*g/m^3^ on cardiovascular mortality was Relative Risk (RR) 1.13 (95% CI 1.09–1.18) and on respiratory mortality was RR 1.03 (95% CI 1.02–1.03) [7]. According to Hoek et al. long-term exposure to NO_2_ increases the risk of death by 5% for every 10 *μ*g/m^3^ NO_2_ [8]. Hamra et al. in their estimates showed that the change in lung cancer incidence or mortality per 10 *μ*g/m^3^ increase in exposure is 4% [95% confidence interval (CI) 1%-8%] [9]. In the following study [10], the authors identified the relationship between NO_2_ and chronic obstructive pulmonary disease (COPD). The pooled effect of a 10 g/m^3^ increase in NO_2_ concentration on hospital admissions and on mortality was 1.3% and 2.6%, respectively. Long-term and short-term NO_2_ exposure on COPD cases had an RR 2.5 and 1.4%, respectively. The COPD effect associated with a 10 *μ*g/m^3^ increase in exposure to outdoor-sourced NO_2_ and to an exclusively traffic-sourced NO_2_ was 1.7 and 17.8%, respectively. According to Brønnum-Hansen et al., reducing NO_2_ exposure to rural levels (6 *μ*g/m^3^) could increase life expectancy by one year in 2040, and 20% reduction in NO_2_ would result in 1.3–1.6 years of disease-free life and 0.3-0.5 years of total life expectancy [11].

Given the aforementioned impacts, scientists and governments have turned their attention to the challenge of reducing NO_2_ emissions. Knowing its concentration in advance can be particularly important for decision-makers when planning and implementing air pollution strategies. The development of new technologies makes it possible to combine the components affecting air pollution, to estimate and forecast them by establishing advanced models.

The model that will be used in this work is Bidirectional convolutional LSTM (BiConvLSTM) to more efficiently capture space-time patterns and make very accurate predictions. Several authors have implemented this model in their work [12, 13], but our study will be the first to implement BiConvLSTM in the air quality domain. Regarding the baseline models, LSTM and ConvLSTM were selected (LSTM—based on the Table 1, which displays publications focusing on NO_2_ prediction with implemented methods extracted from the following work [14]; ConvLSTM—given the fact that many authors have recently used it for air quality prediction). Therefore, the main objective of this work is to predict NO_2_ concentration using BiConvLSTM. The analysis was carried out in two scenarios: a) Including all datasets, and b) Including datasets selected by the implementation of the feature selection technique. Both scenarios were designed to answer the following questions: Compare the selected model (BiConvLSTM) with other models (LSTM-FC, ConvLSTM) for predicting NO_2_ in the city of Madrid in terms of accuracy and runtime. The analysis was implemented using data from Madrid during the period January-June 2019 (training set) and January-June 2020 (validation and testing sets) with the purpose to predict the next 6 hours using the previous 6 hours of data. The main contributions of ongoing work can be summarised as follows: a) The prediction of NO_2_ deploying spatiotemporal method, b) Endorsement of the proposed model’s superiority over the reference models, and c) Emphasis of the advantages of the feature selection method.

**Table 1:** Implemented algorithms and evaluation metrics extracted from the publications focused on the prediction of NO_2_ (*).

Work	ML Algorithm	Evaluation Metric
[15]	BRT, SVM, XGBoost, RF, GAM, Cubist	RMSE, ME, NRMSE, NME, POD, POF, R^2^
[16]	LSTM	RMSE, NSE, PBIAS, R
[17]	LSTM	MSE
[18]	MLR, MLPNN, ELM, OSMLR, OSELM	
[19]	LSTM	RMSE, MAE
[20]	ELM	RMSE, MAE, IA, R^2^
[21]	ANN	RMSE, R, NMB, NMSD, R_s_, SD, SD′
[22]	SVM, M5P model trees, ANN	RMSE, NRMSE, PTA
[23]	Cluster-based bagging	RMSE, R^2^, RMSEIQR
[24]	MLP with hierarchical clustering, SOM and k-means clustering	RMSE, MAE, NRMSE, MBE, IA, R
[25]	GAM, Bagging, RF, GBM, ANN, KRLS, SVR, Linear stepwise regression algorithms, Regularization or shrinkage algorithms	RMSE, R^2^, MSE-R^2^
[26]	Ensemble model with DRR	RMSE
[27]	AIS-RNN (RNN, LSTM, GRU)	RMSE, MAE, MAPE
[28]	SVM	RMSE, MAE, CWIA, RE
[29]	RF partition model	MAPE, MADE, BIC, R^2^
[30]	SVM	RMSE, MAE, WIA
[31]	LSTM	RMSE

* **ML Algorithms**: *BRT*–Boosted Regression Trees, *SVM*–Support Vector Machine, *XGBoost*–EXtreme Gradient Boosting, *RF*–Random Forest, *GAN*–Generalized Additive Model, *LSTM*–Long Short Term Memory, *ANN*–Artificial Neural Network, *GBM*–Gradient Boosting Machines, *KRLS*–Kernel-based Regularized Least Squares, *AIS*–Adaptive Input Selection, *RNN*–Recurrent Neural Network, *GRU*–Gated Recurrent Unit, *MLR*–Multiple Linear Regression, *MLPNN*–Multi-layer Perceptron Neural Networks, *ELM*–Extreme Learning Machine, *OSMLR*–Online Sequential Multiple Linear Regression, *OSELM*–Online Sequential Extreme Learning Machine, *SOM*–Self-organizing Map, *DRR*–Discounted Ridge Regression; Evaluation Metrics: *RMSE*–Root Mean Squared Error, *ME*–Mean Error, *NRMSE*–Normalized Root Mean Squared Error, *NME*–Normalized Mean Error, *POD*–Probability of Detection, *POF*–Probability of False Alarm, *R^2^*–Coefficient of Determination, *NSE*–Nash–Sutcliffe Efficiency Index, *PBIAS*–Percentage Bias, *R*–Pearson Correlation Coefficient, *MSE*–Mean Squared Error, *MAE*–Mean Absolute Error, *IA*–Index of Agreement, *NMB*–Normalised Mean Bias, *R_s_*–Rank Correlation by Spearman, *SD*–Standard Deviation, *PTA*–Prediction Trend Accuracy, *MBE*–Mean Bias Error, *MAPE*–Mean Absolute Percentage Error, *CWIA*–Complementary Willmott’s Index of Agreement, *RE*–Relative Error, *MADE*–Mean Absolute Deviation Error, *BIC*–Bayesian Information Criterion, *WIA*–Willmott’s Index of Agreement.

The rest of the paper is structured as follows. Section 2 is dedicated to identifying related works. Section 3 introduces the case study and describes the datasets employed and the methodology implemented. Section 4 presents the implementation process and the results obtained. Finally, Section 5 includes the conclusions and future work.

## 2 Related work

Predicting air quality is challenging given the numerous factors that affect it. With the development of technologies various models, including statistical and deep learning models, have been deployed to predict air quality. The choice of model can be adjusted depending on the stated problem to be solved, for example, the predicted pollutant or the study region’s peculiarities. Below are a few examples of research related to the subject area extracted from the following works [14, 32].

For example, Xu et al. [33] employed the Extreme Gradient Boosting (XGBoost) integrated with the Shapley additive explanation technique for ultrafine particle concentrations forecast. Another work implemented XGboost was developed by Ma et al. [34] to predict PM_2.5_ in Shanghai. Leong et al. [35] applied Support Vector Machine to predict air pollution index. Lasisi et al. [36] proposed Fuzzy Rough Set and Artificial Immune System algorithms to predict air quality. Among many studies, many of them have confirmed the effectiveness of the Recurrent Neural Network due to the temporal correlation of air quality data. For example, Fong et al. applied Long Short-Term Memory (LSTM) combined with transfer learning and pre-trained neural networks [17] to predict air pollutants in the next day using meteorological and air pollutant’s concentration data of Macau. Zhai and Cheng performed a one-day forecast implementing LSTM on air quality, meteorological and social media data [19]. Another work by Yang et al. proposed hybrid Convolutional Neural Network (CNN)-LSTM and CNN-Gated Recurrent Unit (GRU) models to predict PM_10_ and PM_2.5_ for the next seven days in Seoul using air pollution and meteorological data [37]. Heydari et al. [38] developed hybrid model based on combination of LSTM and multi-verse optimization algorithm to predict the air pollution obtained from Combined Cycle Power Plants (Kerman, Iran).

In addition to forecasting along the time axis, it is also important to consider the spatial dimension, and identify the air quality value in places where there are no stations. Several authors have focused on the spatial factor in their studies. Danesh Yazdi et al. [39] proposed ensemble machine learning based on a Random Forest (RF), a Gradient Boosting Machine (GBM), and a k-nearest Neighbor (KNN) to predict PM_2.5_ using air quality, satellite aerosol optical depth, land use, and meteorological data. Li et al. [23] suggested Kruskal-K-means clustering method to predict NO_2_ and NO_x_. Just et al. [40] applied XGBoost to predict PM_2.5_ using satellite-derived aerosol optical depth integrated with recursive feature selection technique. Zou et al. [41] applied spatiotemporal attention based LSTM on the Beijing dataset. Ma et al. implemented a Bidirectional LSTM (BLSTM) network with Inverse Distance Weighting to predict PM_2.5_ concentration at Guangdong, China [42]. Ma et al. [43] presented Transfer Learning-based Stacked Bidirectional Long Short Term Memory network to predict air quality in Anhui, China. Le et al. [44] implemented Convolutional LSTM (ConvLSTM) to interpolate and predict PM_2.5_ in the city of Seoul. Also, Alléon et al. [45], and Liu and Shuo [46] applied ConvLSTM for forecasting air quality. Phruksahiran implemented the geographically weighted predictor method to predict air quality index in Bangkok and Thailand [47].

## 3 Materials and methods

### 3.1 Study area and data description

**Fig 1:**
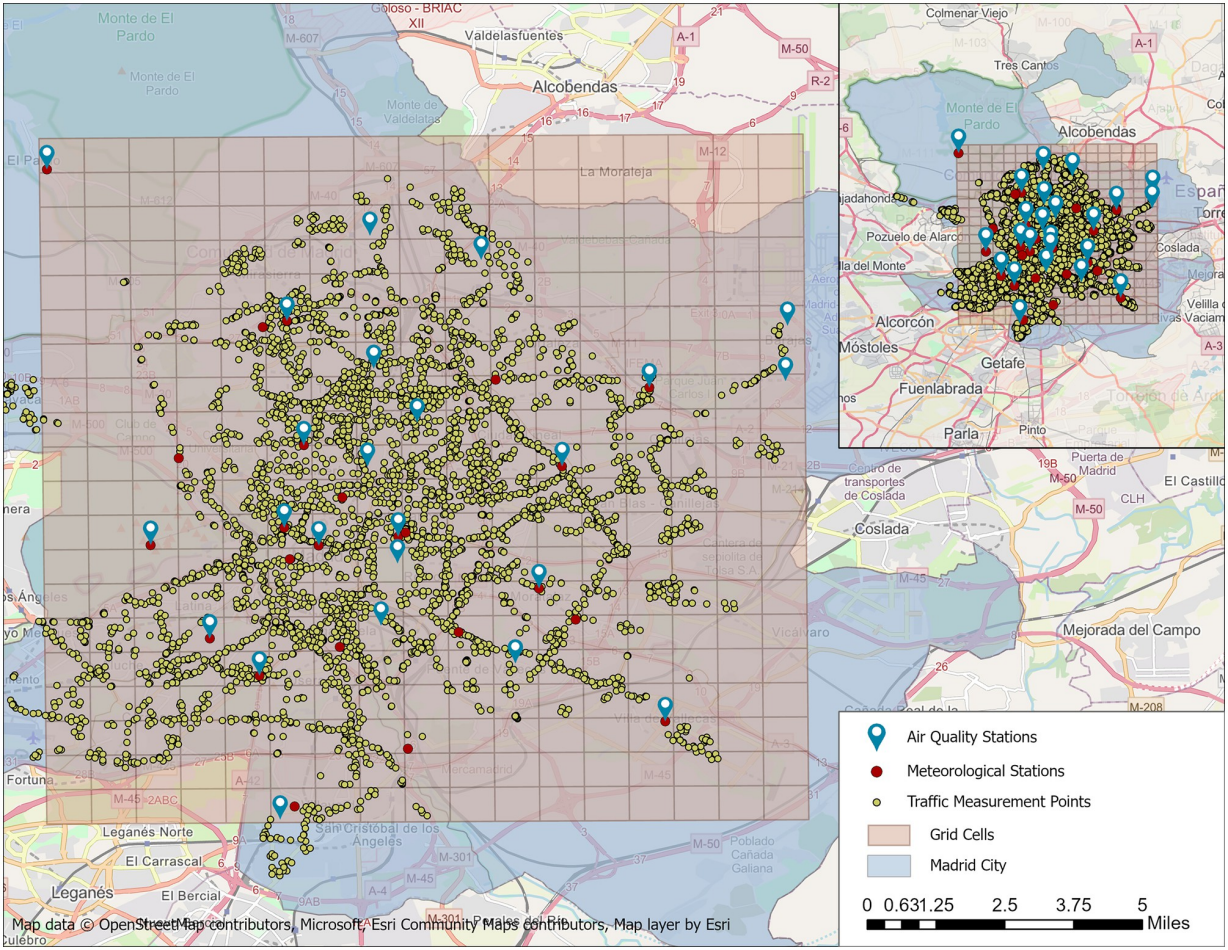
Air quality stations, meteorological stations, traffic measurement points and grid cells segments on the defined area of the city of Madrid (Map data © OpenStreetMap contributors, Microsoft, Esri Community Maps contributors, Map layer by Esri [50]).

The study area considered in this work is the city of Madrid (Fig 1). It has an area of about 604.31 km^2^, and it is the second largest city in the European Union in terms of population (3,305,408 [48]). According to the study by Sasha Khomenko et al. [49] related to premature mortality due to air pollution in European cities, in which the pollutants PM_2.5_ and NO_2_ were considered, Madrid was found to be at the top of the ranking of European cities with the highest NO_2_ mortality burden. Taking into consideration the importance of NO_2_ for Madrid, it was selected as an air pollutant for predictive analysis.

In a study carried out by Cuevas et al. [51] the authors observed the temporal evolution of NO_2_ in five Spanish cities, including Madrid, over the period 1996-2012. Applying the shift trend model to NO_2_ data, they found that NO_2_ levels in the Madrid area had dropped by about 53%. A comparison of average annual values obtained from air quality monitoring stations showed that the decline in Madrid is 37%. This decline is associated with the implementation of environmental policies and technologies, as well as with the consequences of the global economic crisis. The study shows that in the pre-recession period the annual decline was 1.1%, and 7.8% during the economic recession. Therefore, it can be seen that economic and industrial factors significantly affect NO_2_ emissions. According to the work by Izquierdo et al. [52], the implementation of the Madrid City air-quality plan would lead to an annual mean decrease in NO_2_ by 4.0 *μ*g/m^3^ in 2020.

While the implementation of control policies and strategies has a positive impact on reducing air pollution, the problem nevertheless still remains the focus of attention. New technologies can help make better and more efficient decisions. Following the aforementioned belief, this work focuses on NO_2_ prediction in the city of Madrid using machine learning technologies.

According to the following study [14], the publications related to the prediction of air quality using machine learning technologies used more than 26 datasets to supplement air quality data (meteorological, spatial, traffic, social media, etc.). The datasets used in this work are NO_2_ data (*μ*g/m^3^), meteorological data and traffic data from January to June 2019 and from January to June 2020, and the location of the monitoring stations. The data were obtained from *Open Data portal of the Madrid City Council* [53]. There are 24 air quality control stations, 26 meteorological control stations and more than 4000 traffic measurement points (shapefiles of measurement point locations are also provided for each month). The meteorological data include ultraviolet radiation (Mw/m^2^), wind speed (m/s), wind direction, temperature (°C), relative humidity (%), barometric pressure (mb), solar irradiance (W/m^2^) and precipitation (l/m^2^), while the traffic data include intensity, occupancy time, load and average traffic speed. The datasets have an hourly rate. Since the attributes of the traffic data can be specific to a certain area, the following are the selected traffic attributes with their definition for the city of Madrid: *Intensity*—Intensity of the measurement point in a period of 15 minutes (vehicles/hour); *Occupancy time*—Measurement point occupancy time in a period of 15 minutes (%); *Load*—Vehicle loading in a 15-minute period. This is a parameter that takes into account intensity, occupation and capacity of the road and establishes the degree of road use from 0 to 100; and *Average traffic speed*—Average speed of the vehicles in a period of 15 minutes (km/h). Only for M30 intercity measuring points.

From the above definitions it can be seen that the traffic data is recorded every 15 minutes. However, since NO_2_ and meteorological data are at hourly rates, the traffic data were filtered and only hourly records were selected (for example, with entries at 13:00, 13:15, 13:30, 13:45 and 14:00, we simply selected the entries at 13:00 and 14:00 and the same logic was applied for the entire period).

Table 2 shows summary statistics of each type of data (since the location of traffic measurement points changes monthly, summary statistics were calculated based on the part that was used in the analysis). The datasets and the code implemented are available at the following links [53–55].

**Table 2:** Summary statistics of the periods January-June 2019 and January-June 2020 for each data type.

	Descriptors	January-June 2019	January-June 2020
Nitrogen_dioxide	Mean (SD)	36.69 (30.85)	26.03 (25.35)
	Median [Min,Max]	27.0 [0.0, 328]	17.0 [0.0, 326]
Ultrav._rad.	Mean (SD)	15.83 (30.27)	-
	Median [Min,Max]	1.0 [0.0, 199]	-
Wind_speed	Mean (SD)	1.41 (1.11)	1.31 (1.05)
	Median [Min,Max]	1.14 [0.0, 8.75]	1.05 [0.0, 8.97]
Wind_direction	Mean (SD)	167.80 (105.72)	140.82 (98.35)
	Median [Min,Max]	182.0 [0.0, 359]	135.0 [0.0, 359]
Temperature	Mean (SD)	13.38 (8.09)	13.63 (7.6)
	Median [Min,Max]	12.5 [-55.0, 47.3]	12.6 [-55.0, 44.6]
Humidity	Mean (SD)	48.73 (21.60)	60.76 (22.77)
	Median [Min,Max]	47.0 [-25, 100]	62.0 [-25, 100]
Pressure	Mean (SD)	943.3 (34.91)	940.62 (63.28)
	Median [Min,Max]	945.0 [0.0, 962.0]	945.0 [0.0, 1073.0]
Solar_irradiance	Mean (SD)	220.73 (301.06)	191.95 (279.83)
	Median [Min,Max]	11.0 [0.0, 1103.0]	9.0 [0.0, 1113.0]
Precipitation	Mean (SD)	0.03 (0.41)	0.03 (0.27)
	Median [Min,Max]	0.0 [0.0, 30.4]	0.0 [0.0, 13.5]
Intensity	Count_non_zero	885863 (59.98%)	892197 (60.09%)
	Mean (SD)	245.69 (402.73)	161.45 (313.33)
	Median [Min,Max]	63.0 [0.0, 6348.0]	34.19 [0.0, 6588.0]
Occupation	Count_non_zero	845031 (57.21%)	822652 (55.41%)
	Mean (SD)	3.96 (6.36)	2.57 (4.9)
	Median [Min,Max]	0.95 [0.0, 100.0]	0.42 [0.0, 99.0]
Load	Count_non_zero	881500 (59.68%)	884950 (59.60%)
	Mean (SD)	11.65 (14.91)	7.85 (11.75)
	Median [Min,Max]	4.0 [0.0, 100.0]	2.2 [0.0, 100.0]
Average_speed	Count_non_zero	233415 (15.8%)	223052 (15.0%)
	Mean (SD)	4.39 (13.28)	4.04 (12.96)
	Median [Min,Max]	0.0 [0.0, 96.5]	0.0 [-127.0, 127.0]

**Fig 2:**
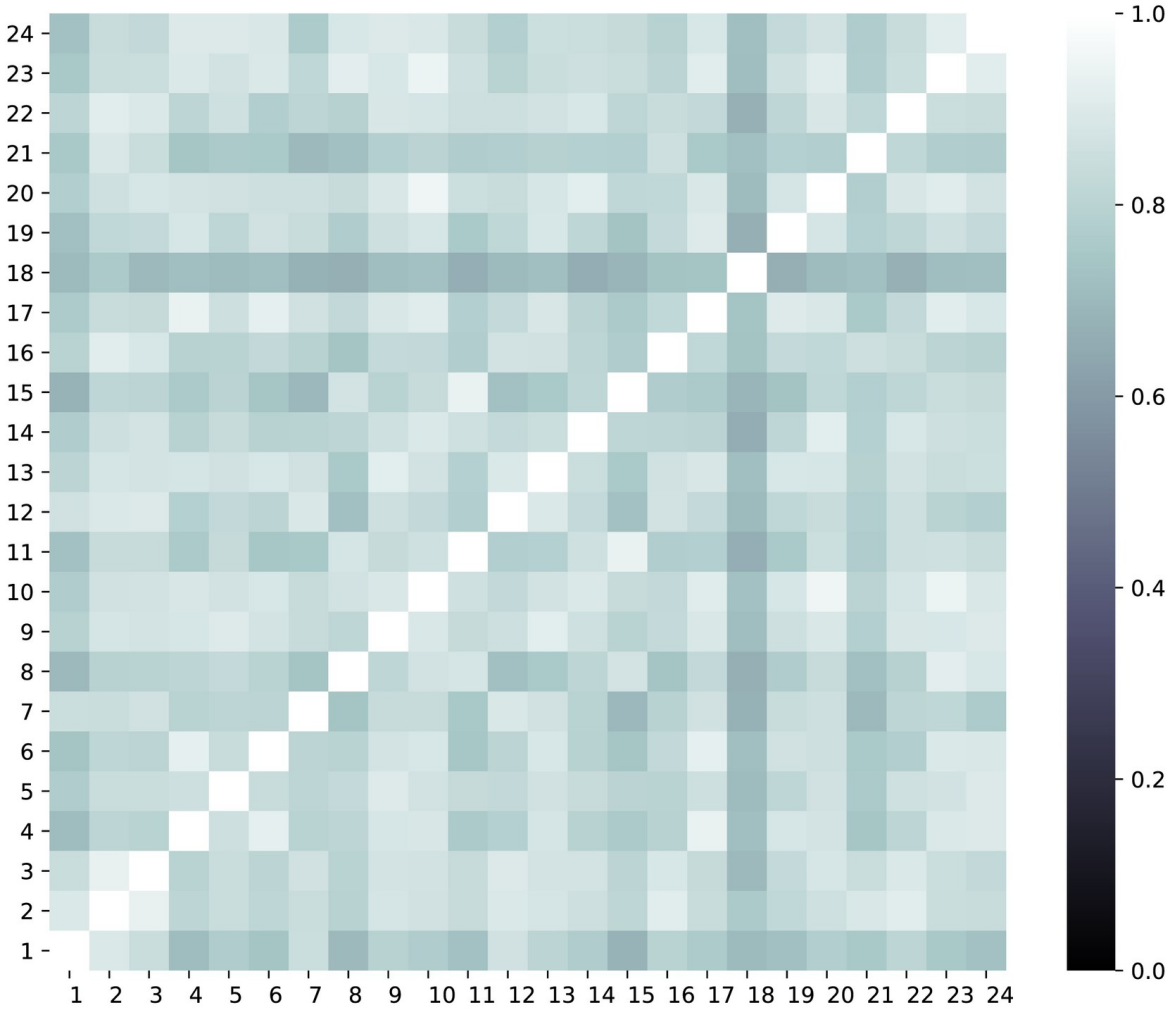
Heatmap showing spatial correlations of the 24 air quality monitoring stations.

**Fig 3:**
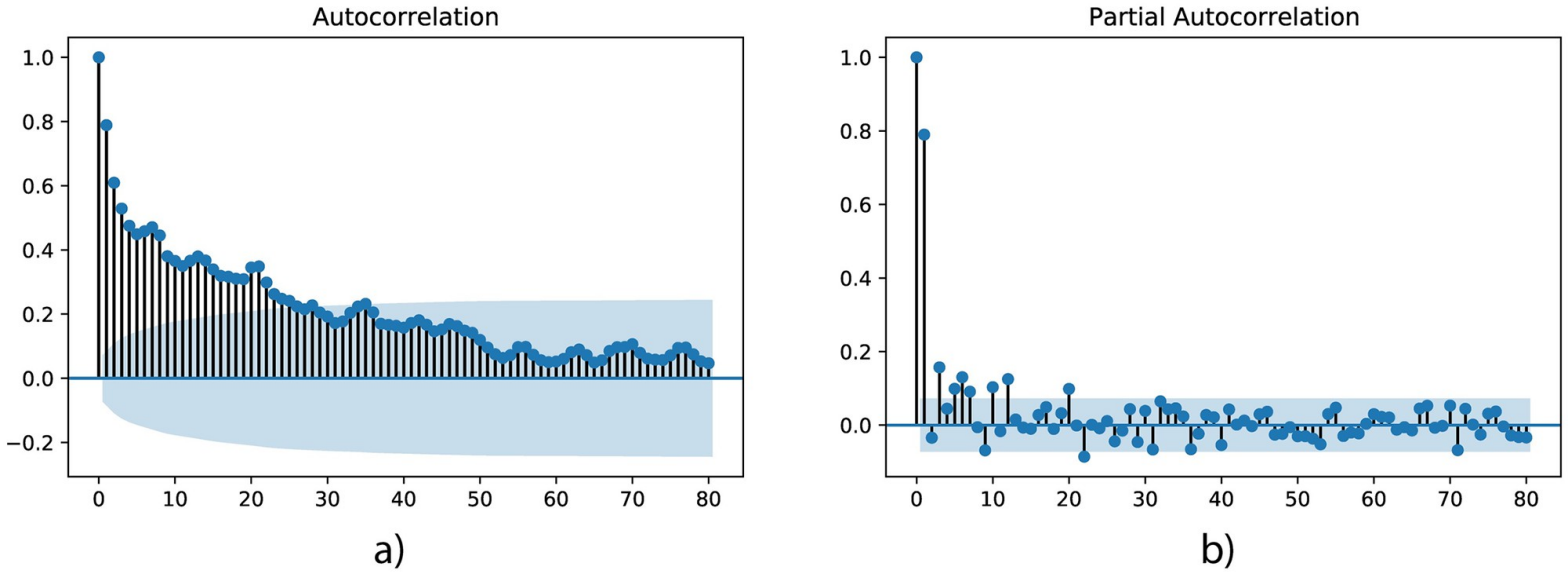
a) Autocorrelation and b) Partial autocorrelation plots with 80 lags from the NO_2_ dataset.

Considering the spatial factor in air quality prediction, the Pearson correlation coefficients between stations were calculated (Fig 2). It can be noticed that the stations are spatially correlated. Fig 3 shows autocorrelation (or the correlogram, the correlation between values of the same series at different time steps) and partial autocorrelation plots of NO_2_ concentration; the daily interval is chosen as a lag length and the plots show the results of 80 lags. The difference between autocorrelation and partial autocorrelation is that in the first case, it calculates the correlation between two lags, taking into account the influence of previous observations (direct and indirect affects), and in the case of partial autocorrelation, it is just a real correlation between two lags without intervening observations (only direct effects). These functions help to determine the best lags, which can be selected for effective forecasting. It can be seen that in the autocorrelation plot more than 25 lags have a significant positive correlation, although if we look at the partial autocorrelation plot, there is a statistically significant correlation for lag 1 and 2 periods. In this work, 6-hour lag was chosen, which are in the range of significant correlated lags.

**Fig 4:**
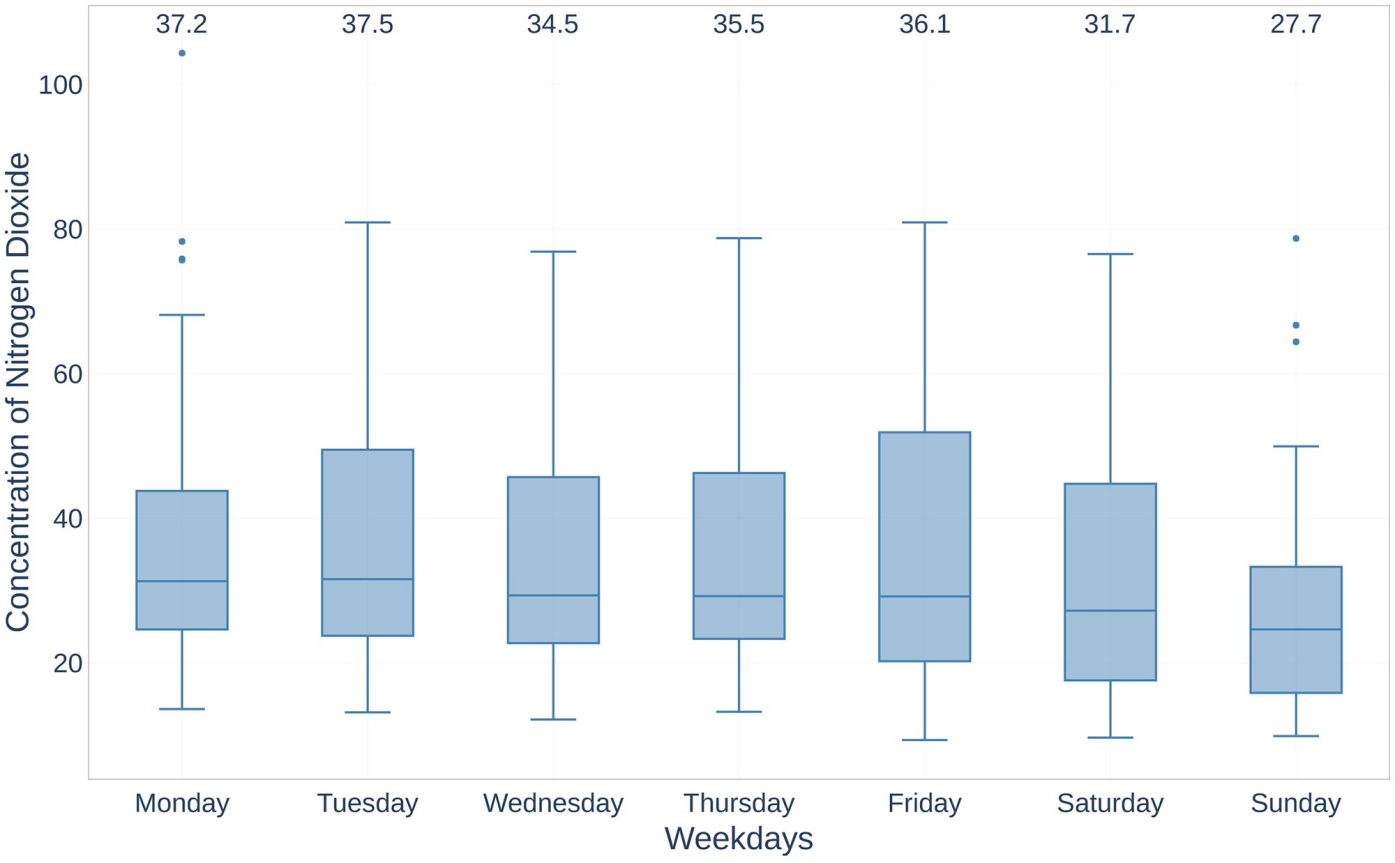
The concentration of NO_2_ in weekdays dimension for the period January-June 2019.

Another interesting observation can be seen in Fig 4, which shows NO_2_ concentration during different weekdays for the period of 2019 using boxplots (the numbers at the top of Fig 4 are mean values corresponding to each boxplot). The concentration distribution can be explained by the traffic factor, which plays a decisive role in raising the level of NO_2_. This recent belief was also confirmed by the following study [56], which showed that in Madrid up to 90% of NO_2_ comes from local traffic.

### 3.2 Method

**Fig 5:**
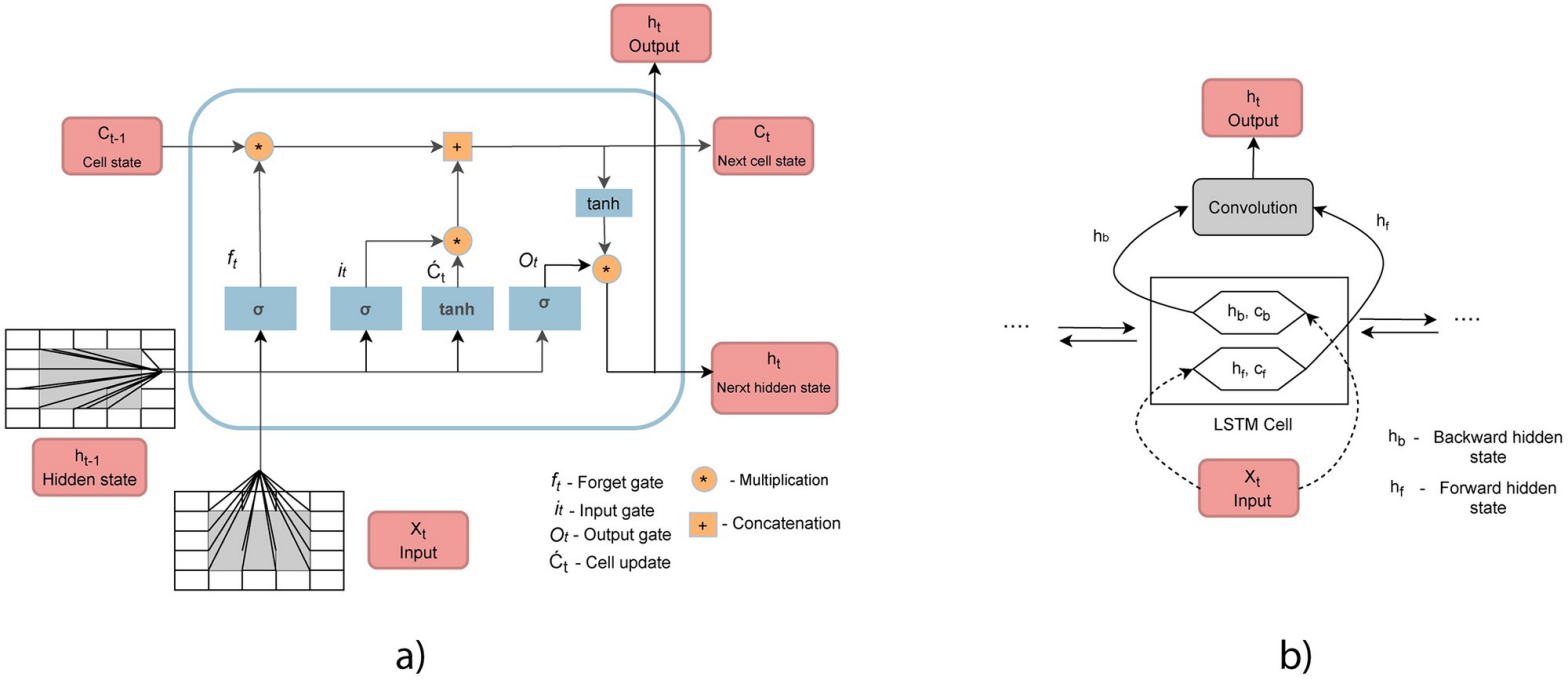
a) The architecture of a ConvLSTM cell [57] and b) Bidirectional ConvLSTM cell [12].

The algorithm that was used in this work is Bidirectional convolutional LSTM (BiConvLSTM). It is an advanced version of ConvLSTM in which hidden and cell states are kept for forward and backward sequences. Fig 5 shows the architecture of (a) ConvLSTM and (b) BiConvLSTM cells. ConvLSTM was first used by Shi et al. [57], who showed that it is possible to preserve spatial information in an LSTM implementation by converting internal matrix multiplication into convolution operations. This spatiotemporal factor, combined with a bidirectional factor, allows for an increased ability to capture more information in the temporal dimension. The hidden states from forward and backward sequences are combined and then go through a convolution layer. There are several ways to execute the combination process (sum, calculate the average, multiply or concatenate), which as a parameter has to be defined during the tuning process (the parameter optimisation is presented in the next section).

Firstly, the ConvLSTM can be formulated with the following equations [57, 58]:

$$\begin{array}{c} \begin{array}{c} i_{t}=\sigma \left(W_{i}^{X}*X_{t}+W_{i}^{H}*H_{t-1}\right)\\ f_{t}=\sigma \left(W_{f}^{X}*X_{t}+W_{f^{H}}*H_{t-1}\right)\\ o_{t}=\sigma \left(W_{o}^{X}*X_{t}+W_{o}^{H}*H_{t-1}\right)\\ C_{t}=f_{t}⊗C_{t-1}+i_{t}⊗tanh(W_{c}^{X}*X_{t}\\ +W_{c}^{H}*H_{t-1})\\ H_{t}=o_{t}⊗tanh\left(C_{t}\right) \end{array} \end{array}$$ (1)

where *i*_*t*_ is the input gate, *f*_*t*_ is the forget gate, and *o*_*t*_ is the output gate (these gates control the flow of information through the cell), *W* is the weight matrix in the forward ConvLSTM cell, *X*_*t*_ is the current input data, *h*_*t*−1_ is previous hidden output, *C*_*t*_ is the cell state, “*” represents the convolution operation and “⊗” represents the Hadamard product. It can be seen that ConvLSTM takes into account only information from past sequences, however combining information from both forward and backward sequences may give better results. Below is the mathematical expression of BiConvLSTM [58].

$$\begin{array}{c} Y_{t}=\text{tanh}\left(W_{y}^{Hf}*H_{t}^{f}+W_{y}^{Hb}*H_{t-1}^{b}\right) \end{array}$$ (2)

where *H*^*f*^ is hidden state from forward ConvLSTM unit, *H*^*b*^ is hidden state from backward ConvLSTM unit, and *Y*_*t*_ is the final output.

## 4 Experiments and results

### 4.1 Experimental settings

**Fig 6:**
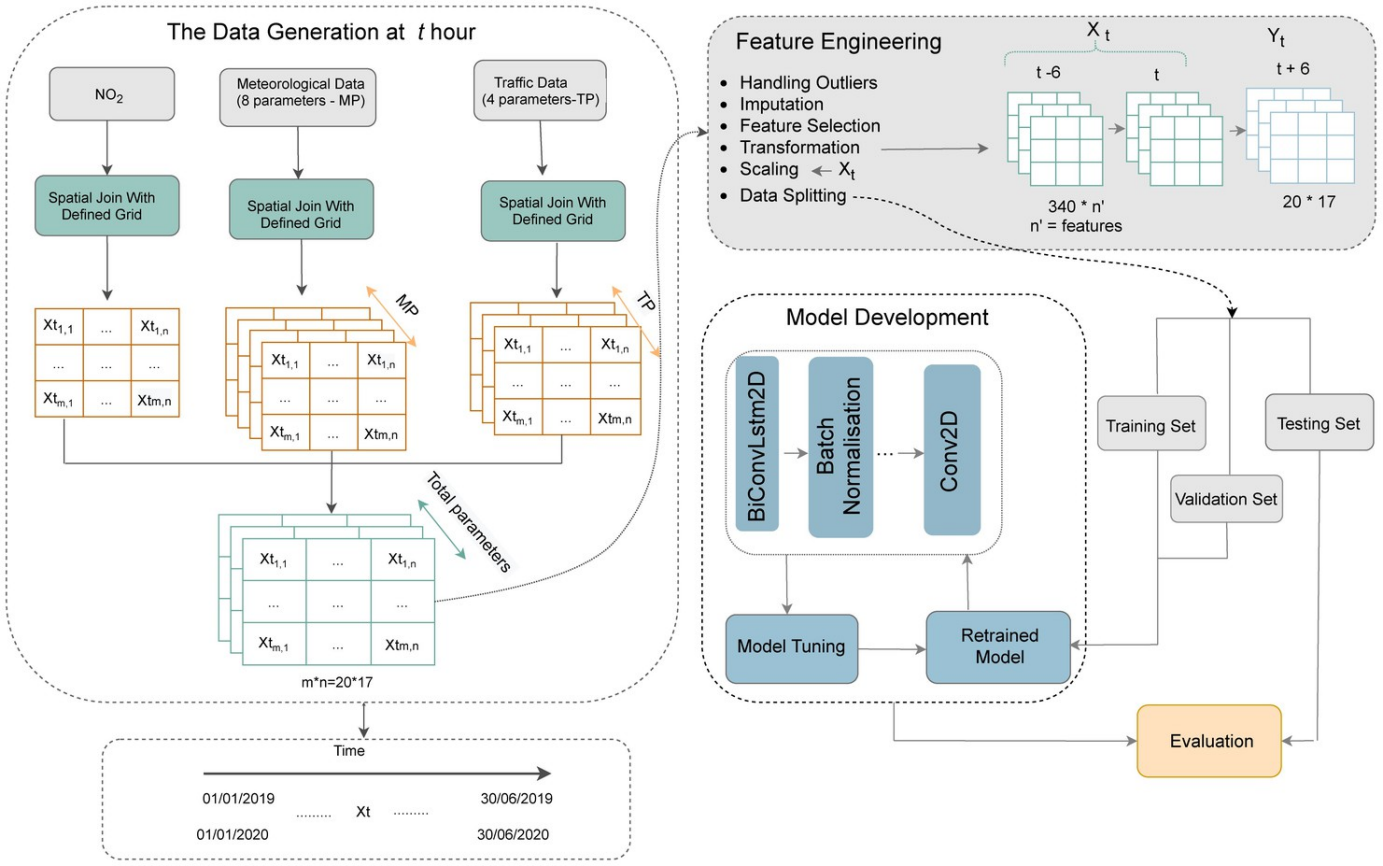
The detailed workflow of the analysis.

This section includes a detailed description of the workflow. The main goal of the current work is to predict NO_2_ in the next 6 hours over a given area, which was carried out based on the data on the previous 6 hours. The overall workflow of the analysis is presented in Fig 6. It can be seen that the workflow consists of the following steps: *Data Generation*, *Feature Engineering*, *Model Development* and *Evaluation*. In terms of tools, ArcGIS Pro software [59] and Google Colab cloud service [60] (with GPU enabled for Pro version) were used to accomplish the proposed tasks.

#### 4.1.1 Data generation

As already mentioned, the raw data was obtained from *Open Data portal of the Madrid City Council* [53]. Since the monitoring stations and measurement points are different for each dataset, the first task is to combine them spatially and temporally. Therefore, the grid was created in a given area, which was defined as a selected part of Madrid with a width and height of 1,000 metres within the following extent: Top—4,486,449.725263 metres; Bottom –4,466,449.725263 metres; Left—434,215.234430 metres; Right –451,215.234430 metres. It was created using ArcPy package [61], specifically the *CreateFishnet* function [62]. There are total of 340 cells (20 by 17) which cover 340 km^2^ or 56.27% of the total area of the city of Madrid. The logic behind selecting this area was to have a minimum extent to include all air quality control stations with the aim of obtaining higher accuracy. The value of each cell includes the values of NO_2_, meteorological and traffic attributes obtained from assigned stations at a certain time. The value of the cells that do not include any stations was assigned as zero and in the case of more than one station, an average value was assigned. The above procedure was repeated for every hour of the selected period. The following functions were used to execute aforementioned process, including *arcpy.management.AddField* [63], *arcpy.analysis.SpatialJoin* [64], *arcpy.da.SearchCursor* [65], *arcpy.da.UpdateCursor* [66]. The output was exported as Comma Separated Values (CSV) files, which were used as an input in further stages of the analysis. Overall, 4344 and 4368 CSV files were generated corresponding to every hour during January-June 2019 and January-June 2020, respectively. A formal description of the data generation process is given by Algorithm 1.

**Algorithm 1** Data generation

**Input**: Data—[Hourly NO_2_, Meteorological and Traffic data]; Period -[01.01.2019-30.06.2019; 01.01.2020-30.06.2020]

1: **for** each hour ∈ Period

2:  Create grid with Fishnet tool (ArcPy library)

3:  Add field to the Fishnet

4:  **for** each item *i* ∈ Data **do**

5:   *i* spatial join with grid

6:   input the mean of the values of each corresponding cell to the field

7:  **end for**

8: **end for**

**Output**: CSV files for each hour including NO_2_, Meteorological and Traffic data

#### 4.1.2 Feature engineering

After generating input data, the next step is feature engineering, which includes the following substeps: *Handling Outliers*, *Imputation*, *Feature Selection*, *Transformation*, *Scaling* and *Data Splitting*.

*4.1.2.1 Handling outliers*. Outliers can reduce the accuracy of the model. Therefore, it is important to process them. Looking at the summary statistics in Table 2, it can be seen that the minimum humidity and temperature values are outliers. Temperatures below -3° for 2019 and -2° for 2020 [67] and humidity with negative values were considered outliers and replaced with the average of the previous and the following values.

*4.1.2.2 Imputation*. This technique was applied to handle missing values of meteorological data. Since meteorological data do not change dramatically within space, we have implemented Nearest Neighbour Interpolation [68].

*4.1.2.3 Feature selection.* The presence of many features sometimes prevents a model from generalising data efficiently, due to the curse of dimensionality. Hence, feature selection must be implemented to select the best combination of datasets, which in turn will prompt the model to efficiently generalise the data. First of all, the following variables were excluded for future predictive analysis: average traffic speed, traffic load, UV, precipitation. Average traffic speed was excluded because it is available only for M30 road which is 15.8% of the case study (Table 2). Traffic load, according to the definition is the combination of intensity, occupancy time and capacity of the road. Therefore, this variables also was excluded, taking into account the fact that it is correlated with other variables. Regarding UV, it was observed that June of 2019 and the whole period of 2020 do not have records about UV. Regarding precipitation, it was found out that around 99% of data were 0, so this feature was also eliminated. Afterwards, the mutual information (*MI*) technique was implemented [69] on the remaining features. It calculates the mutuality between additional datasets and the target dataset (NO_2_). The formula to calculate mutual information is presented below (Eq (3)).

$$\begin{array}{c} \begin{array}{c} MI\left(x;y\right)=\;\int \int \;P\left(x_{i},y\right)log\frac{P(x_{i},y)}{P\left(x_{i}\right)P\left(y\right)}\;dx_{i}\;dy\\ =H(x)-H(x|y) \end{array} \end{array}$$ (3)

where *P*(*x*_*i*_, *y*) is the joint probability distribution of two variables, *P*(*x*_*i*_) and *P*(*y*) are marginal distributions, *H*(*x*) is the entropy for x, and *H*(*x*|*y*) is the conditional entropy.

**Fig 7:**
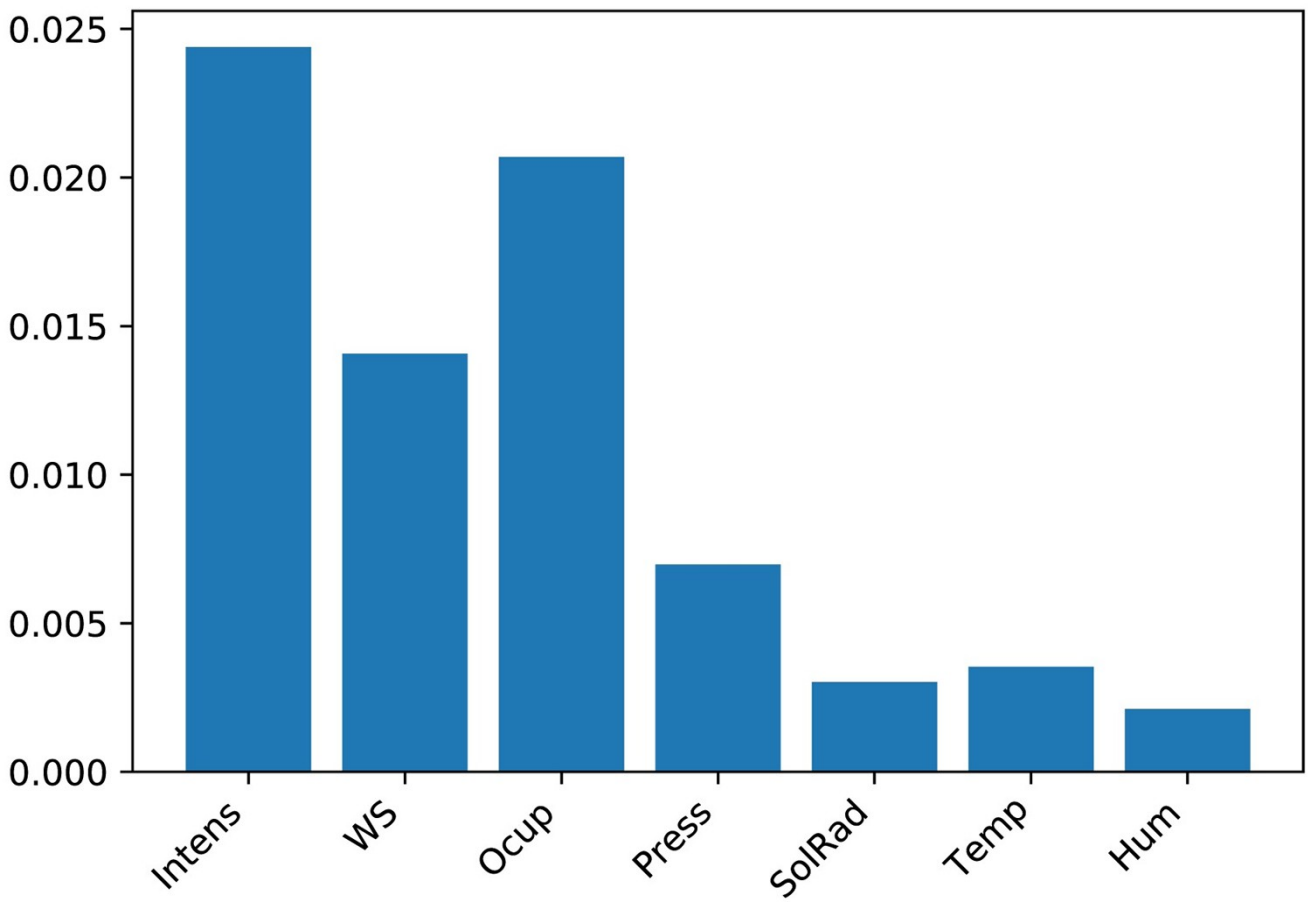
The feature importance scores based on mutual information.

Fig 7 shows the feature importance scores of 7 additional datasets based on mutual information. For further analysis in the second scenario, features with a score above 0.005 were selected, including wind speed, barometric pressure, intensity and occupancy time. It should be mentioned that wind direction also was selected considering the interconnection with wind speed. The reason for not including wind direction in the mutual information calculation process is that the wind direction is circular data and needs to be converted for later use (details below).

*4.1.2.4 Transformation*. In this step wind direction was converted in categorical data with the following categories: north, east, south, west, southwest, northeast, southeast, northwest, and later by implementing One Hot Encoder [70] it was included in the analysis. Another transformation was the conversion of the input data into the supervised learning dataset. Independent and dependent datasets were generated based on the defined time granularity (to predict NO_2_ in the next 6 hours on the basis of data for the previous 6 hours).

*4.1.2.5 Scaling*. Scaling is a very useful technique for handling differences that exist between ranges of the features. The current work applied Min-Max (0-1) normalisation in order to normalise the input data (Eq (4)).

$$\begin{array}{c} X_{norm}=\frac{X-X_{min}}{X_{max}-X_{min}} \end{array}$$ (4)

*4.1.2.6 Data splitting*. After preprocessing the data with the above-mentioned techniques, the next step is to split the dataset into training, validation and testing sets. The data was splitted with the following order: January-June 2019—training set; January-March 2020—validation set; April-June 2020—testing set. The dimension of each sets is illustrated in Table 3.

**Table 3:** The dimension of each sets.

Set	Dimension (*x* × *y* × *z*_1_/*z*_2_)(*)
Training Set	4344 × 340 × 16/13
Validation Set	2184 × 340 × 16/13
Testing Set	2184 × 340 × 16/13

* *x*—Number of samples; *y*—Number of grid cells (340 = 20×17); *z*_1_—Number of all features (NO_2_, wind speed, temperature, humidity, barometric pressure, solar irradiance, intensity, occupancy time, north, east, south, west, southwest, northeast, southeast, northwest), *z*_2_—Number of selected features (NO_2_, wind speed, barometric pressure, intensity, occupancy time, north, east, south, west, southwest, northeast, southeast, northwest). Note that features include wind directions after the implementation of One Hot Encoder.

#### 4.1.3 Model development

This step presents the process of model construction. The parameter optimisation of the proposed model was performed by applying GridSearchCV with Blocking Time Series Split. Blocking Time Series Split was chosen instead of cross-validation because it considers the time series aspect and prevents leakage from one set to another. In order to reduce the computation time for parameter optimisation, GridSearchCV was applied on data for one month. Table 4 shows optimised parameters with the options that were tried, and the one that was finally selected is indicated in bold.

**Table 4:** Parameter optimisation with GridSearchCV.

Parameters	Options
Number of Filters	8, **16**, 32
Kernel Size	**(3,3)**, (5,5), (7,7), (9, 9)
Optimiser	RMSprop, **Adam**
Merge Mode	‘**concat**’, ‘mul’, ‘sum’, ‘ave’
Number of Layers	2, **3**, 4

Therefore, the architecture of the model was built based on the chosen parameters by stacking 3 bidirectional ConvLSTM layers with a kernel size of 3x3 (it should be noted that a model with a smaller kernel allows capturing slower motion), filters equal to 16 and with an Adam optimiser. It can be seen that concatenation was selected as the merge mode, which means that the forward and backward ConvLSTM units were concatenated before passing information to the next unit. Each BiConvLSTM layer was followed by Dropout and Batch Normalization layers, and the model was finalised using a 1x1 convolution layer.

Regarding the baseline models, LSTM-FC had the following structure: 2 LSTM layers with 2048 units followed by Dropout layer and the model was finalised adding a Dense layer; ConvLSTM had 5x5 kernel size with filters equal to 32, followed by Batch Normalisation and Dropout layers and it was finalised with 1x1 convolution layer.

#### 4.1.4 Evaluation

After parameter optimisation the finalised model was evaluated in the testing set in order to answer the questions defined in the Introduction. From Table 1, it can be seen that RMSE and MAE are the most used evaluation metrics, therefore, these metrics were chosen as evaluation metrics. RMSE measures the geometric difference between estimated and actual values and it is very sensitive to large errors (Eq (5)), and MAE measures the average magnitude of the errors (Eq (6)).

$$\begin{array}{c} RMSE={\left(\frac{1}{n}\sum_{i=1}^{n}{\left(E_{i}-A_{i}\right)}^{2}\right)}^{1/2} \end{array}$$ (5)

$$\begin{array}{c} MAE=\frac{1}{n}\sum_{i=1}^{n}|E_{i}-A_{i}| \end{array}$$ (6)

where *n* is the number of instances, and *E*_*i*_ and *A*_*i*_ are the estimated and actual values. The lower the value is, the better the prediction will be. Algorithm 2 provides pseudo code of NO_2_ prediction procedure.

**Algorithm 2** NO_2_ prediction

**Input**: CSV files for each hour including NO_2_, Meteorological and Traffic data

**function** calculate Nearest Neighbour Interpolation(Meteorological data)

2:  **return** *zero values of meteorological data impute by Nearest Neighbour interpolation*

  **end function**

4: **function** Handling Outliers (data)

  **return** *outliers converted to the average of the previous and the next non outliers*

6: **end function**

 **function** Data Splitting(data)

8: **return** *independent and dependent data split based on time resolution*

  **end function**

10: Split data on training, validation and testing sets with the following order: January-June 2019—training sets; January-March 2020—validation sets; April—June 2020—testing set

Normalise input set

12: Reshape data based on selected model architecture

 **function** Create Model (model parameters by default)

14: **return** *model architecture*

 **end function**

16: **function** GridSearchCV(parameters to tune)

  **return** *best parameters*

18: **end function**

 **function** evaluate model (model with best parameters)

20:  **return** *error estimated with evaluation metric*

 **end function**

 **Output**: RMSE, MAE

### 4.2 Results and discussion

As mentioned in the Introduction, the analysis was carried out according to two scenarios. Below are the results for each of them.

#### 4.2.1 First scenario

In this scenario the experiments were performed using 9 features (NO_2_, wind speed, wind direction, temperature, relative humidity, barometric pressure, solar irradiance, intensity, and occupancy time) without the remaining 4 features (UV, precipitation, load and average traffic speed), which, as mentioned above, were excluded immediately after the data exploration phase, given the obvious reasons for the exclusion. Table 5 presents the results obtained and the runtime of the models for the next 6-hour lag. Looking at the results of the RMSE and MAE, it can be seen that BiConvLSTM outperforms ConvLSTM and LSTM-FC with values of 19.14 and 13.06, respectively. In particular, in terms of RMSE, BiConvLSTM improves results compared to ConvLSTM by 41.9%, and to LSTM-FC by 50.8%. in terms of MAE, BiConvLSTM improves results compared to ConvLSTM by 59.24%, and to LSTM-FC by 59.4%. Regarding runtime, due to the complexity of the BiConvLSTM architecture, it takes a comparably longer time for the model to converge.

**Table 5:** Prediction errors (RMSE, MAE) and runtime of the models for the next 6 hours prediction implemented on all features.

Models	RMSE (*μ*g/m^3^)	MAE (*μ*g/m^3^)	Time
LSTM-FC	38.89	32.17	4m15s
ConvLSTM	32.95	32.04	33m15s
BiConvLSTM	19.14	13.06	36m57s

#### 4.2.2 Second scenario

In this scenario, the analysis was carried out using the datasets selected after calculating the feature importance scores based on mutual information. Table 6 shows RMSE and MAE values and runtime of the models performed on the selected features. It can be seen that, as in the first scenario, in this case also BiConvLSTM surpassed other models. Especially, in terms of RMSE, BiConvLSTM improves results compared to ConvLSTM by 16.28%, and to LSTM-FC by 19.32% in terms of MAE, BiConvLSTM improves results compared to ConvLSTM by 18.32%, and to LSTM-FC by 28.21%. Regarding runtime, in this case also BiConvLSTM converges comparably slower than ConvLSTM and LSTM-FC.

**Table 6:** Prediction errors (RMSE, MAE) and runtime of the models for the next 6 hours prediction implemented on the selected features.

Models	RMSE (*μ*g/m^3^)	MAE (*μ*g/m^3^)	Time
LSTM-FC	15.68	13.54	3m58s
ConvLSTM	15.11	11.9	27m53s
BiConvLSTM	12.65	9.72	34m33s

The difference between the two scenarios, which can be observed, is a significant decrease of the values in terms of runtime and the error, which is associated with the peculiarities of the implementation of the feature selection methodology. It is essential to understand why, among all the features, only some of them (wind speed, wind direction, barometric pressure, intensity, and occupancy time) were chosen, what is the relationship between NO_2_ and features with a higher mutual information index, the inclusion of which as a result improved the performance of the model. In terms of wind speed and direction, the correlation is because an increase in wind speed suggests a lower concentration due to increased dilution through advection and increased mechanical turbulence. In terms of traffic data, the transport sector has been confirmed to be one of the largest sources of nitrogen oxides (nitrogen oxide and NO_2_), for example, about 46% of total emissions in 2013 in the European Union were attributed to nitrogen oxides [71].

It is worth to mention that the units of RMSE and MAE are defined in the same unit as the target variable; therefore, in the current work, it matches with the unit of NO_2_ (μg/m^3^). Hence, by looking at the results, it can be seen that MAE is 9.72 μg/m^3^, which can be considered sufficient comparing with mean values of NO_2_ (36.69 and 26.03 for the period 2019 and 2020, respectively). It is essential to consider the impact of the Coronavirus Disease 2019 (COVID-19) during 2020 to combat some measures, such as traffic restrictions and self-isolation, and as a result, these events have affected the air pollution concentration. In the case of Madrid, due to COVID-19 restrictions, the concentration of NO_2_ dropped to 62% [72]. These sudden changes can also affect the model’s performance, and it would be ideal for future work to compare the results with a different period to identify these effects.

Overall, it can be seen that BiConvLSTM outperforms other reference models in both scenarios; however, regarding the execution time, it takes comparable more time. The superiority of the proposed model over LSTM can be explained by the fact that BiConvLSTM captures spatial information, while LSTM focuses exclusively on temporal information. Compared to ConvLSTM, the advantage of existing forward and backward sequences of BiConvLSTM helps to collect more information and, as a result, outperforms ConvLSTM. On the other hand, these sequences lengthen the execution time.

## 5 Conclusions and future work

Taking into account the impact of NO_2_ on health and the environment, the management and control of its value become an essential issue for governments and decision-makers (according to WHO guidelines, NO_2_ has the following threshold values: 40 *μ*g/m^3^ and 200 *μ*g/m^3^, respectively, for the annual average and for the 1-hour average [6]). Considering that the concentration of NO_2_ correlates both temporally and spatially, this work implements BiConvLSTM, which can perform effectively in temporal and spatial dimensions. The data used for analysis are NO_2_, meteorological and traffic data from January to June 2019 and from January to June 2020 in the city of Madrid. Two scenarios were developed based on the subsets of features used in the analyses. The proposed model was compared to ConvLSTM and LSTM-FC, and the results showed that BiConvLSTM outperformed the reference models in both scenarios. In particular, feature selection improved the final results by 33.9% in terms of RMSE and by 25.27% in terms of MAE. Regarding runtime, BiConvLSTM is slower due to the model architecture, and it takes longer to converge the data. Moreover, the output showed that the feature selection step is important because it significantly reduces the error. It is worth noting that by looking at the results of the MAE and comparing them with the average concentration values, the proposed model can be considered a reliable and robust model.

As regards the limitations, it is worth mentioning that the predictive analysis was performed using Google Colab, and the cloud service itself has restrictions in terms of the amount of data and the complexity of the model [73]. However, with access to a more powerful machine learning analysis platform, the scale of optimisation of the parameters of the proposed model could be expanded, more data could be generated and included in the training set, and perhaps the performance of the model could be improved. In terms of the proposed model’s limitations, the requirement of the input data, which is related to the model’s architecture, can be specified. As it can be seen, the input data must be in grid format. However, grid formatting can be challenging, since in the case of lack of data, modification of the original data will be required, which may have an impact on the model performance. Therefore, another machine learning model, such as a graph neural network, could be developed in the future, with the results of alternative approaches compared. Other aspects that could be considered as future work may be the integration of other datasets, such as street networks and buildings, application the proposed procedure to a different pollutant (for example, for PM_2.5_ as it has serious health effects), as well as to other cities in order to compare performance based on spatial characteristics. Also, as already mentioned, it would be ideal for performing analysis for a different period and observing the impact of COVID-19 on the model’s execution.
